# Evaluating the Reparative Potential of Secretome from Patient-Derived Induced Pluripotent Stem Cells during Ischemia–Reperfusion Injury in Human Cardiomyocytes

**DOI:** 10.3390/ijms251910279

**Published:** 2024-09-24

**Authors:** Elise Rody, Jeremy Zwaig, Ida Derish, Kashif Khan, Nadezda Kachurina, Natalie Gendron, Nadia Giannetti, Adel Schwertani, Renzo Cecere

**Affiliations:** 1 Department of Surgery, Division of Cardiac Surgery, McGill University Health Center, Montreal, QC H4A 3J1, Canada; 2 Faculty of Medicine, McGill University, Montreal, QC H3G 2M1, Canada; jeremy.zwaig@mail.mcgill.ca (J.Z.);; 3 Department of Surgical and Interventional Sciences, McGill University, Montreal, QC H3G 1A4, Canada; 4 Department of Medicine, Division of Cardiology, McGill University Health Center, Montreal, QC H4A 3J1, Canadanadia.giannetti@mcgill.ca (N.G.);

**Keywords:** secretome, proteomics, induced pluripotent stem cells, ischemia–reperfusion, cardiomyocytes, dilated cardiomyopathy, cell-free therapeutics

## Abstract

During a heart attack, ischemia causes losses of billions of cells; this is especially concerning given the minimal regenerative capability of cardiomyocytes (CMs). Heart remuscularization utilizing stem cells has improved cardiac outcomes despite little cell engraftment, thereby shifting focus to cell-free therapies. Consequently, we chose induced pluripotent stem cells (iPSCs) given their pluripotent nature, efficacy in previous studies, and easy obtainability from minimally invasive techniques. Nonetheless, using iPSC secretome-based therapies for treating injured CMs in a clinical setting is ill-understood. We hypothesized that the iPSC secretome, regardless of donor health, would improve cardiovascular outcomes in the CM model of ischemia–reperfusion (IR) injury. Episomal-generated iPSCs from healthy and dilated cardiomyopathy (DCM) donors, passaged 6–10 times, underwent 24 h incubation in serum-free media. Protein content of the secretome was analyzed by mass spectroscopy and used to treat AC16 immortalized CMs during 5 h reperfusion following 24 h of hypoxia. IPSC-derived secretome content, independent of donor health status, had elevated expression of proteins involved in cell survival pathways. In IR conditions, iPSC-derived secretome increased cell survival as measured by metabolic activity (*p* < 0.05), cell viability (*p* < 0.001), and maladaptive cellular remodelling (*p* = 0.052). Healthy donor-derived secretome contained increased expression of proteins related to calcium contractility compared to DCM donors. Congruently, only healthy donor-derived secretomes improved CM intracellular calcium concentrations (*p* < 0.01). Heretofore, secretome studies mainly investigated differences relating to cell type rather than donor health. Our work suggests that healthy donors provide more efficacious iPSC-derived secretome compared to DCM donors in the context of IR injury in human CMs. These findings illustrate that the regenerative potential of the iPSC secretome varies due to donor-specific differences.

## 1. Introduction

Induced pluripotent stem cells (iPSCs) revolutionized the field of regenerative medicine after their breakthrough discovery by Yamanaka et al. in 2006 [1]. These “miracle cells” have multiple advantages, possessing embryonic-like qualities and being easily expandable. IPSCs can be derived from a range of non- or minimally invasive sources, including urine, peripheral blood, skin fibroblasts, etc. [1,2,3,4,5,6,7]. In addition, their use has steadily increased in popularity due to the iPSCs’ potential to maintain donor characteristics. For instance, iPSC-derived cardiomyocytes (CMs) originating from donors with arrhythmia presented with the same arrythmias [8,9,10]. These characteristics make them attractive cell types for regenerative applications and in vitro disease modelling.

Many challenges have arisen since the inception of iPSCs, such as high tumorigenicity upon implantation, the need for differentiation protocol development, and limitations in the creation of high-throughput methodologies. The potential for undifferentiated cells to remain during implantation and become tumorigenic has largely halted transplantation progress until better differentiation protocols can be developed. To this end, CM differentiation from iPSCs remains a contested point with no universally accepted protocol [8,9,10]. All existing protocols faced the same challenges when it came to obtaining high numbers of pure iPSC-CMs and greater difficulty in obtaining a mature cell phenotype as characterized by the disappearance of spontaneous beating and aligned actinin rods [11,12,13,14]. The heart is complex, containing over 2 billion heart muscle cells; producing sufficient CMs to provide effective treatment has also proven challenging.

Although the last 20 years have seen much clinical research utilizing stem cells for mediating cardiac repair, the results have been disheartening and unreliable. Many phase I and II clinical studies utilizing administration of mesenchymal stem cells (MSCs) or other cardiac stem cells during reperfusion procedures in ischemic hearts have failed to obtain results needed to justify significant financial investment in phase III clinical trials [15,16,17,18,19,20,21,22,23,24,25]. Additionally, most of the beneficial effects observed were attributed to the factors secreted by implanted stem cells, as the cells were often cleared from the site of injury, even though their positive effects were still observed [15,16,17,18,19,20,21,22,23,24,25].

The cell secretome is the ensemble of factors composed of extracellular vesicles, proteins, ribonucleic acid (RNA), and lipids. It is multi-purposed, facilitating waste disposal, cell-to-cell communication, and even inter-organ communication. Extracellular vesicles can also contain proteins, RNA, and lipids and are resistant to premature degradation of bioactive particles. Early studies have even shown that extracellular vesicles are more effective at locating the site of injury when peripherally injected compared to stem cells, which tend to locate in the liver or lungs [26,27,28], thereby empowering minimally invasive techniques over highly invasive direct injections. Additionally, findings repeated by many groups suggest that MSC-derived secretomes can have pro-angiogenic effects on endothelial cells and immunomodulatory functions on macrophages [26,27,28,29,30]. These characteristics make secretome an attractive option for regenerative-based therapies.

In this study, we generated iPSCs from CD34+ cells obtained from dilated cardiomyopathy (DCM) patients, the most common type of cardiomyopathy [31,32], and healthy donors to treat an in vitro disease model of ischemia–reperfusion (IR) injury. We hypothesized that the iPSC-derived secretome, regardless of donor health status, would provide therapeutic benefit when administered during reperfusion following ischemia.

## 2. Results

### 2.1. iPSC-Derived Secretome from Healthy Donors Are Enriched with Proteins Involved in Calcium Handling Pathways

**Figure 1:**
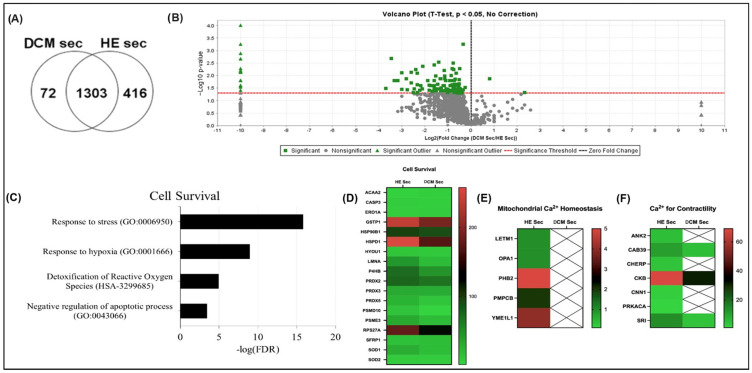
Proteomic profiling of iPSC-derived secretome from healthy donors and dilated cardiomyopathy patients. (**A**) Venn diagram comparing identified proteins in secretome. (**B**) Volcano plot identifying differentially expressed proteins. (**C**) GO pathway analysis of iPSC secretome from all iPSC lines. (**D**) Heatmap of differentially expressed proteins involved in cell survival, (**E**) mitochondrial Ca^2+^ homeostasis, and (**F**) intracellular Ca^2+^ and contractility. Data are presented in number of peptides. DCM sec = dilated cardiomyopathy patient-derived secretome; HE sec = healthy donor-derived secretome.

Proteomic content of collected secretome found 1791 proteins with 72 expressed exclusively in the secretome of DCM-derived iPSC lines and 416 exclusively expressed in healthy donor-derived stem cell lines (Figure 1A). Of the 1303 proteins expressed in both DCM patient-derived secretome and healthy donor-derived secretome, *t*-tests determined differential expression of 139 proteins with mainly higher expression of proteins in healthy donor-derived secretome (Figure 1B). STRING pathway analysis [33] found 391 proteins contributing to pathways responding to stress, hypoxia, negative regulation of apoptotic process, and detoxification of reactive oxygen species (ROS) (Figure 1C). Notably, 19 were involved in at least three of these four pathways with no significant differences in relative expression between the healthy and DCM donor-derived iPSC secretome (*p* > 0.05; Figure 1D). STRING pathway analysis [33] also revealed a decrease in five proteins associated with mitochondrial calcium homeostasis pathways in iPSC-derived secretome from DCM patients (*p* < 0.05) compared to healthy donors (Figure 1E). Another seven proteins involved in intracellular calcium homeostasis, calcium signalling, and regulation of muscle contractions were differentially expressed in secretomes from healthy donors compared to DCM donors (*p* < 0.05; Figure 1F).

### 2.2. iPSC-Derived Secretome Increased CM Viability after IR Injury

**Figure 2:**
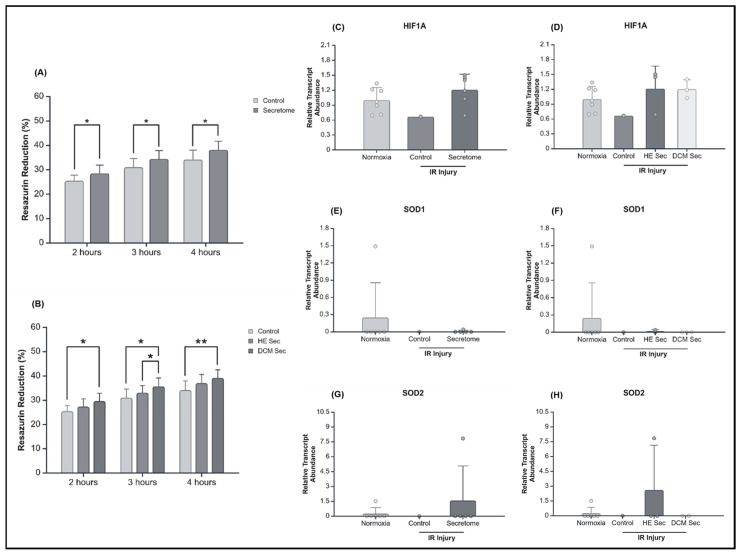
Effect of secretome on viability as measured by metabolic activity after hypoxia-reperfusion injury. Percentage of resazurin reduction at 2, 3, and 4 h post-ischemia–reperfusion injury comparing control to secretome (**A**) grouped (*n* = 1 with six repeats and 6 with six repeats each, respectively), and (**B**) separated based on donor health status (*n* = 1 with six repeats, with six repeats, and 3 with six repeats). Relative transcript abundance of Hypoxia inducible factor subunit 1 alpha with secretome (**C**) grouped (*n* = 7, 1, and 6), and (**D**) ungrouped (*n* = 7, 1, 3, and 3); of superoxide dismutase 1 with secretome (**E**) grouped (*n* = 6, 1, and 5), and (**F**) ungrouped (*n* = 6, 1, 2, and 3); and of superoxide dismutase 2 with secretome (**G**) grouped (*n* = 6, 1, and 5), and (**H**) ungrouped (*n* = 6, 1, 2, and 3). * *p* < 0.05, ** *p* < 0.01. HE Sec = healthy donor secretome; DCM Sec = dilated cardiomyopathy donor secretome; HIF1A = hypoxia inducible factor subunit 1 alpha; SOD = superoxide dismutase; IR = ischemia–reperfusion. Relative abundance was calculated by normalization of raw quantitative data against the mean of the normoxia-treated group.

The previously validated model consisting of 24 h of hypoxia followed by 5 h of reperfusion was used for this disease modelling study [34], wherein AC16 CMs were incubated with secretome from individual donor lines or complete media during reperfusion. Cell survival was rescued by secretome treatment (*p* < 0.05) as measured by an increase in resazurin reduction by metabolically active cells (Figure 2A,B). DCM donor-derived secretome-treated cells tended to have increased resazurin reduction compared to control media condition. At 2 h post-reperfusion the observed an increase in resazurin reduction by the DCM group compared to healthy donors tended towards significance (*p* = 0.081). At 3 h, there was a significant increase in resazurin reduction by the DCM group compared to the healthy donor group (*p* < 0.01). However, by hour four, post-reperfusion, the difference in resazurin reduction was no longer significant (*p* = 0.163). To this effect, we looked at hypoxia inducible factor 1 subunit alpha (HIF1A) and superoxide dismutase (SOD) 1 and 2. HIF1A has been demonstrated to play a role in adapting tissue to low oxygen conditions, whereas SOD1 and -2 have been found to have a role in cardioprotection against ROS reduction [35,36]. Therefore, all three genes play a role in reducing metabolic stress and DNA damage and may reduce apoptosis. We did not, however, observe any differences in their relationship between treatment groups (*p* > 0.05; Figure 2C,E,G) or in function of donor health status (*p* > 0.05; Figure 2D,F,H).

**Figure 3:**
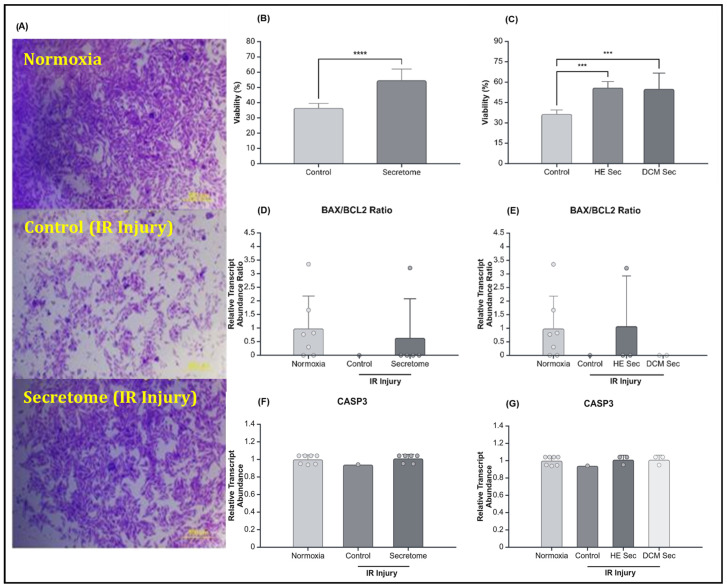
Effect of secretome on cell survival and relative transcript abundance of genes related to apoptosis. (**A**) Representative pictures of AC16 cardiomyocytes at 4× magnification dyed with crystal violet prior to solubilization with methanol after being cultured in normoxia conditions or after ischemia–reperfusion injury with control media treatment or secretome treatment wherein purple represents viable cells still attached to the plate. Percentage of viability following ischemia–reperfusion injury comparing control to secretome (**B**) grouped (*n* = 1 with six repeats and 6 with six repeats each, respectively) and (**C**) ungrouped (*n* = 1 with six repeats, 3 with six repeats each, and 3 with six repeats each). Relative transcript abundance of B-cell lymphoma 2-associated X/B-cell lymphoma 2 ratio with secretome (**D**) grouped (*n* = 7, 1, and 5), and (**E**) ungrouped (*n* = 7, 1, 3, and 2); and of caspase 3 with secretome (**F**) grouped (*n* = 7, 1, and 6), and (**G**) ungrouped (*n* = 7, 1, 3, and 3). *** *p* < 0.001, **** *p* < 0.0001. HE Sec = healthy donor secretome; DCM Sec = dilated cardiomyopathy donor secretome; BCL2 = B-cell lymphoma 2; BAX = B-cell lymphoma 2-associated X; CASP3 = caspase 3; IR = ischemia–reperfusion. Relative abundance was calculated by normalization of raw quantitative data against the mean of the normoxia-treated group.

We further assessed cell survival via the crystal violet viability assay immediately following reperfusion. Congruently to the results obtained during resazurin reduction assay, we observed that cell viability was increased in the secretome treatment (*p* < 0.0001–0.001; Figure 3B,C). However, there was no statistically significant difference in B-cell lymphoma 2-associated X (BAX)/B-cell lymphoma 2 (BCL2) ratio or in relative transcript abondance of Caspase3 (CASP3) between treatment groups (*p* > 0.05; Figure 3D,F), or in terms of donor health (*p* > 0.05; Figure 3E,G).

**Figure 4:**
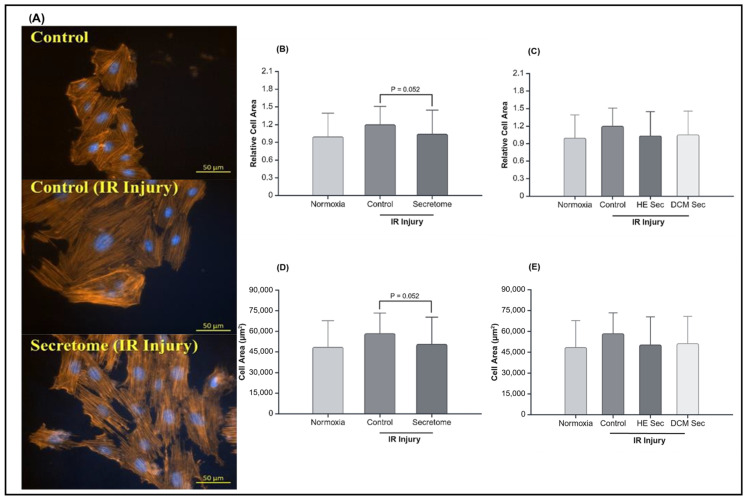
Effect of secretome on the hypertrophic response after ischemia–reperfusion injury. (**A**) Representative pictures of AC16 CMs with the nucleus and actin-dyed. Relative cell area following ischemia–reperfusion injury comparing control to secretome (**B**) grouped (*n* = 7 with 3–4 repeats, *n* = 1 with 36 repeats, and *n* = 6 with 47–77 repeats), and (**C**) ungrouped (*n* = 7 with 3–4 repeats, *n* = 1 with 36 repeats, *n* = 3 with 47–77 repeats, and *n* = 3 with 55–77 repeats). Cell area following ischemia–reperfusion injury comparing control to secretome (**D**) grouped (*n* = 7 with 3–4 repeats, *n* = 1 with 36 repeats, and *n* = 6 with 47–77 repeats), and (**E**) ungrouped (*n* = 7 with 3–4 repeats, *n* = 1 with 36 repeats, *n* = 3 with 47–77 repeats, and *n* = 3 with 55–77 repeats). HE Sec = healthy donor secretome; DCM Sec = dilated cardiomyopathy donor secretome; IR = ischemia–reperfusion.

Finally, cells were assessed for hypertrophy, a typical sign of maladaptive remodelling following IR injury. There was tendency towards a statistically significant reduction in hypertrophy following secretome treatment (*p* = 0.052; Figure 4B,D). Upon separating the secretome group based on donor health status, we no longer observed significant differences between groups (*p* > 0.05; Figure 4C,E). Additionally, we looked at the relative abundance of atrial natriuretic peptides (ANP) and brain natriuretic peptides (BNP), genes related to hypertrophy and mechanical stress but found no detectable levels in the 1 million cells assessed by quantitative reverse transcription polymerase chain reaction.

### 2.3. iPSC-Derived Secretome from Healthy Donors Improved Calcium Contractility

**Figure 5:**
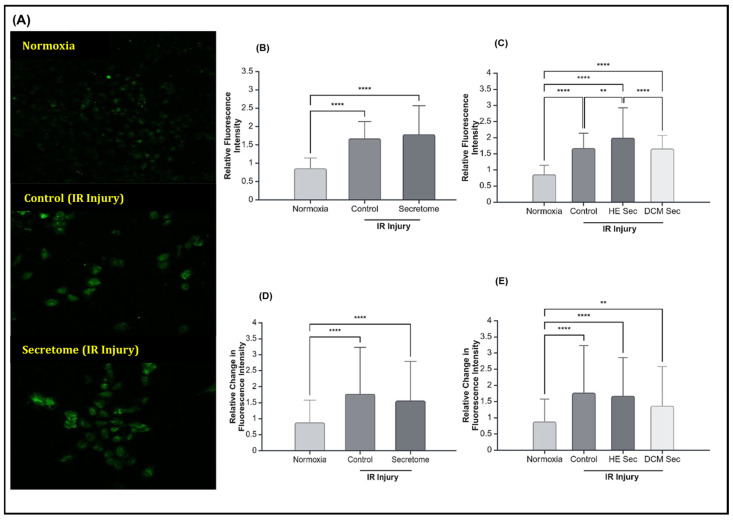
Effect of secretome on calcium handling after hypoxia–reperfusion injury. (**A**) Representative fluorescence imaging at 20× magnification of cardiomyocytes where calcium was stained with Fluo-4, AM, after being cultured in normoxia conditions or after ischemia–reperfusion with control media treatment or secretome treatment. Relative intracellular calcium fluorescence intensity in cardiomyocytes treated with secretome (**B**) grouped (*n* = 7 with 28–101 repeats, *n* = 1 with 54 repeats, and *n* = 6 with 27–83 repeats), and (**C**) ungrouped (*n* = 7 with 28–101 repeats, *n* = 1 with 54 repeats, *n* = 3 with 27–43 repeats, and *n* = 3 with 33–83 repeats). Relative change in intracellular calcium fluorescence intensity in cardiomyocytes treated with secretome (**D**) grouped (*n* = 7 with 28–101 repeats, *n* = 1 with 58 repeats, and *n* = 6 with 28–29 repeats), and (**E**) ungrouped (*n* = 7 with 28–101 repeats, *n* = 1 with 58 repeats, *n* = 3 with 32–33 repeats, and *n* = 3 with 24–25 repeats). ** *p* < 0.01, **** *p* < 0.0001. HE Sec = healthy donor secretome; DCM Sec = dilated cardiomyopathy donor secretome; IR = ischemia–reperfusion.

**Figure 6:**
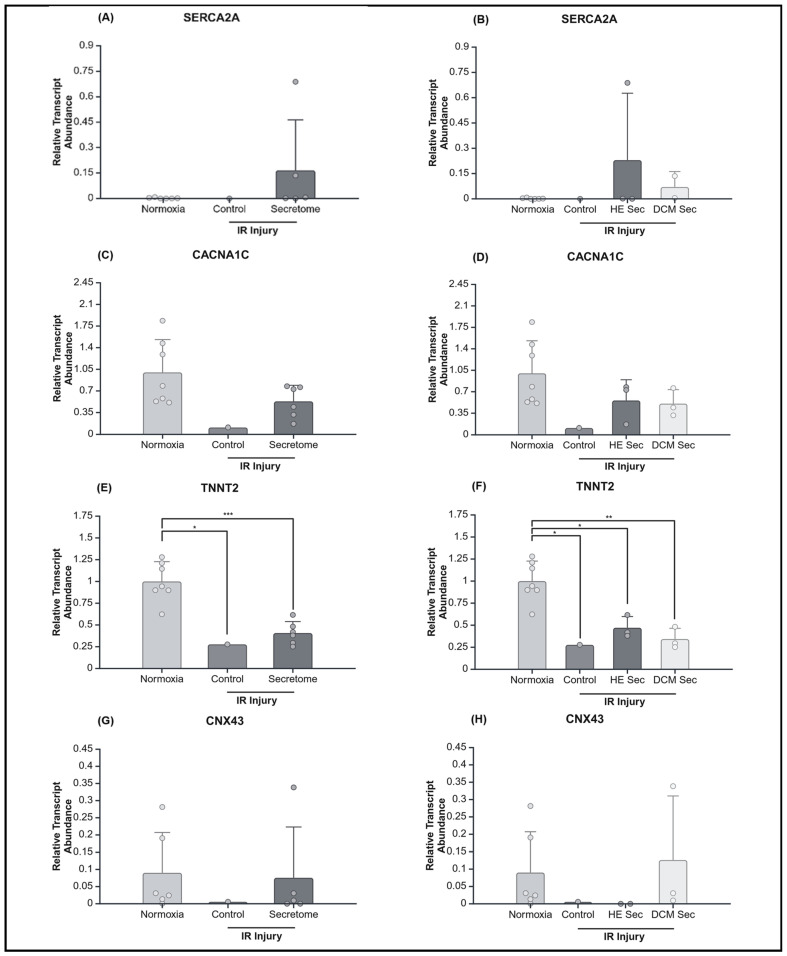
Relative abundance transcript abundance of genes involved in calcium contractility. Transcripts identified have various roles relating to contractility and calcium homeostasis and hypoxia response. Relative transcript abundance of sarcoplasmic/endoplasmic reticulum Ca^2+^ ATPase 2a with secretome (**A**) grouped (*n* = 6, 1, and 5), and (**B**) ungrouped (*n* = 6, 1, 3, and 2); of calcium voltage-gated channel subunit alpha 1 C, with secretome (**C**) grouped (*n* = 7, 1, and 6), and (**D**) ungrouped (*n* = 7, 1, 3, and 3); of Cardiac troponin T 2 with secretome (**E**) grouped (*n* = 7, 1, and 6), and (**F**) ungrouped (*n* = 7, 1, 3, and 3); and of connexin 43 with secretome (**G**) grouped (*n* = 6, 1, and 5), and (**H**) ungrouped (*n* = 6, 1, 2, and 3). * *p* < 0.05, ** *p* < 0.01, *** *p* < 0.001. HE Sec = healthy donor secretome; DCM Sec = dilated cardiomyopathy donor secretome; SERCA2A = sarcoplasmic/endoplasmic reticulum Ca^2+^ ATPase 2a; CACNA1C = calcium voltage-gated channel subunit alpha 1 C; TNNT2 = cardiac troponin T 2; CNX43 = connexin 43; IR = ischemia–reperfusion. Relative abundance was calculated by normalization of raw quantitative data against the mean of the normoxia-treated group.

Although IR injury caused the expected increase in mean and change in calcium fluorescence intensity, iPSC secretome treatment did not improve calcium contractility; neither in terms of mean fluorescence intensity (*p* > 0.05; Figure 5B) nor change in fluorescence intensity (*p* > 0.05; Figure 5D). We observed that only treatment with healthy donor-derived secretome lead to increases in mean calcium fluorescence intensity (*p* < 0.01; Figure 5C) but not in relative change in calcium fluorescence intensity (*p* > 0.05; Figure 5E). Accordingly, we looked at relative transcript abundance of genes relating to intracellular calcium homeostasis, calcium signalling, and the regulation of muscle contractions. We found TNNT2 to be significantly downregulated by IR injury (*p* < 0.05) but there were no significant differences in terms of treatment or donor health status (*p* > 0.05; Figure 6E,F). There was no effect of treatment or donor health status for sarcoplasmic/endoplasmic reticulum Ca^2+^ ATPase 2a (SERCA2A; *p* > 0.05; Figure 6A,B), calcium voltage-gated channel subunit alpha 1 C (CACNA1C; *p* > 0.05; Figure 6C,D), or connexin 43 (CNX43; *p* > 0.05; Figure 6G,H). There were no detectable levels of ryanodine receptor 2 (RYR2) or sodium/calcium exchanger 1 (NCX1) in the AC16 CMs.

## 3. Discussion

In this study, we evaluated the reparative potential of donor-specific iPSC-derived secretomes for the purposes of IR injury. Proteomic analysis of iPSC-derived secretome from healthy donors showed enriched pathways involved in mitochondrial calcium homeostasis and calcium contractility compared to secretomes from iPSCs of DCM patients. We then assessed the therapeutic and regenerative effects of the secretome using a previously validated model of IR injury in human CMs [34]. Collected iPSC secretome via 24 h conditioned media, a method of incubating cells with extracellular vesicle-depleted media, was used to treat ischemia-injured AC16 CMs during the 5 h reperfusion period. This allowed us to evaluate the iPSC-derived secretome as a therapeutic option.

Over the last 20 years, a panoply of stem cell-based clinical trials that have largely failed to achieve long-term engraftment and observed beneficial effects were attributed to the stem cell secretome rather than the cells themselves [15,16,17,18,19,20,21,22,23,24,25]. These observations created the opportunity to shift the focus from stem cells to cell-free therapies, which have low immunogenicity [37], easily modifiable content [27,30,38,39,40], an ability to localize to cite of injury [26,27,28], and are obtainable from multiple cell sources. Many studies have focused on comparing secretome content in terms of original localization of a single cell type [28,41,42,43,44,45]. For instance, in 2018, Kehl D et al. [28] found that MSCs from umbilical cord tissue contained more pro-angiogenic factors than MSCs from adipose tissue. Conversely, adipose tissue MSC-derived secretomes had increased pro-proliferative factors when compared to their counterparts originating from umbilical cords [28]. To date, there is limited research on undifferentiated iPSC-derived secretome content or donor characteristics, wherein many groups have chosen to look at iPSC-CM secretomes from healthy donors. One study analyzing donor characteristics found that horse MSC secretome content was affected by age, wherein quantity of proteins in MSC-derived secretome generally decreased with age, but that sex had no effect on secretome content [45]. In our study, utilizing sex-matched donors who were mostly of Caucasian descent, the secretome-derived from the healthy group contained higher levels of mutually expressed protein and had a higher total number of proteins expressed. The healthy donor population was slightly older at 49 ± 4.6 years compared to the 40.6 ± 3.1 years of age in the DCM population. In humans, aging is very heterogenous [46,47], but it is possible that more differences in protein expression could have been observed in a perfectly age-matched population.

Despite this, we observed significant increases in iPSC secretome content of proteins involved in mitochondrial calcium homeostasis, intracellular calcium homeostasis, calcium signalling, and regulation of muscle contractions in healthy donor-derived secretome compared to DCM secretome. These observations were supported by increases in mean calcium florescence intensity when comparing AC16 CMs treated with healthy donor iPSC secretomes and AC16 CMs treated with DCM donor iPSC secretomes.

Increased intracellular calcium concentrations can lead to increases in contractile force via the binding of calcium to TNNT. Relative transcript abundance of TNNT2 was significantly reduced by IR injury. Intracellular calcium concentration increased as a result of IR injury. Due to the additional increase observed in AC16 CMs treated with healthy donor secretome compared to DCM-derived secretome-treated or control media-treated AC16 CMS and the observed increases in secretome content in proteins related to calcium contractility, we hypothesize that this may be a sign of adaptive remodelling to increase contractile force rather than being a maladaptive response. Another mechanism for CMs to increase contractile force is by relying on cell hypertrophy to thicken the ventricular wall [48]. However, chronic hypertrophy of cells can lead to the thinning of the myocardial wall characteristic of DCM [48]. Following secretome treatment, we saw a reduction in hypertrophy, possibly caused by the observed reduction in cell death, limiting the need for CM remodelling. To this effect, there were no observable transcript levels of ANP or BNP. ANP has an anti-hypertrophic effect [49,50], and we would have expected it to have been increased in the secretome-treated AC16CMs if maladaptive remodelling techniques were mediated by the secretome. On the other hand, we would have expected BNP transcript levels to be increased in the control treated groups if hypertrophic response was becoming maladaptive due to the increased stretch of CMs.

Conversely, elevated intramitochondrial calcium concentrations can induce apoptosis [51]. Another possible explanation is that there may have been an increase in intramitochondrial calcium concentration in the control and DCM-treated conditions, not measured in the scope of this project. This hypothesis is consistent with the decreases in cell survival observed in the control group but not for the DCM-derived secretome-treated groups, which largely saw increases in cell survival similar to the healthy donor secretome-treated groups. Despite elevated levels of CASP3 proteins in iPSC secretome content, we did not observe differences in transcript levels of CASP3 as a measure of AC16 CM susceptibility to apoptosis following IR regardless of treatment or donor health status. Similarly, BCL2 and BAX were not affected by treatment or donor health status. We did not evaluate the apoptotic status of AC16 CMs after IR injury. The difference in the crystal violet and resazurin reduction assays allow us to estimate that up to 19% of AC16 CMs treated with secretome derived from healthy donor iPSCs and 16% for the DCM treated group may have been apoptotic, as apoptotic cells are less metabolically active. This was not statistically significant.

Finally, increases in calcium can cause calcium overload [52,53], which can generate ROS [54,55], one of the main mediators of cell damage during IR injury [56]. In the iPSC secretome, regardless of donor health status, we found elevated protein levels of SOD 1 and 2, which have been found to play a role in cardioprotection against ROS reduction [35,36]. This may have played an important role in mitigating cell death as observed in AC16 CMs treated with secretome during IR injury, as evidenced by increased cell survival. Yet, SOD1 and 2 transcript abundance in AC16 CMs was not affected by treatment of donor health status.

Calcium overload can also lead to electrophysiological dysregulation. Notably, healthy donor secretomes contain many proteins involved in calcium contractility, including protein kinase cAMP-activate catalytic subunit A (PRKACA). Protein kinase increases SERCA2A sensitivity to calcium and plays an important role as a contractility moderator [57]. In turn, SERCA2A allows the transportation of calcium ions from the cytoplasm to the sarcoplasmic reticulum and plays an integral role in preventing calcium overload [57]. We found SERCA2A transcript abundance to be unaffected by IR injury or donor health status. Healthy donor iPSC secretome also contained increases in ankyrin 2 proteins. Ankyrin 2 plays an integral part in the assembly and stability of the sodium/calcium exchanger (NCX). NCX can respond quickly to changes in intracellular concentrations of sodium and calcium and has the ability for bi-directional exchange [57]. There were no detectable transcript levels of NCX1. We would have expected to see an increase in NCX1 in healthy donor-derived iPSC secretome consistent with the increased ankyrin 2 protein levels and increased intracellular calcium concentrations.

The lack of IR injury follow-up protein assays anchors the suggested mechanisms of action firmly in the realm of speculation. However, we hypothesize that the results from such follow-up assays would further confirm our findings rather than provide new information. We believe that proteins involved in cell survival would not yield statistically significant results and that proteins involved in mitochondrial calcium homeostasis and calcium contractility would be significantly more elevated in AC16 CMs treated with healthy patient-derived iPSC-secretome compared to their DCM counterparts. This study would therefore require more in-depth analysis of CM electrophysiology to benefit the most from the results issuing from protein follow-up testing. Additionally, other limitations of the study could be addressed beforehand.

For instance, the utilization of AC16 CMs as a disease model represents an important limitation in this study. AC16 CMs are an immortalized line. Immortalized cells have been modified to improve proliferative capabilities [58], which is inaccurate when compared to CMs, which have a regenerative rate declining from 1% during adult life [59]. By using an in vitro cell model that has not been modified in this way or even an in vivo model, we may have observed an increase in the effect size of iPSC-derived secretome treatments and increased the statistical power of our study. Indeed, iPSC secretome-derived extracellular vesicles from a healthy donor were used to treat an unrelated iPSC-CM line and were found to increase survival after IR injury [60]. To our knowledge, we are the first to evaluate the effects of iPSC-derived secretome on calcium handling in CMs in vitro. However, others have found that iPSC or iPSC-CM secretomes improve LVEF following IR injury in mice [61,62,63,64,65].

Additionally, secretome includes extracellular vesicles, RNAs, and lipids, in addition to proteins. Extracellular vesicles have mostly been studied in the context of their content and ability to localize to the site of injury. However, extracellular vesicle treatment has been found to have a cardio-protective effect and improve cardiovascular outcomes, such as LVEF, in in vivo studies utilizing mouse models [25,26,27,66]. RNAs have been studied for their ability to effect changes at both the level of the transcriptome and the proteome [30,67]. Extracellular vesicles-derived from iPSC-CMs have been found to contain RNAs linked to angiogenesis, anti-apoptosis, cardiac regeneration, etc. [66]. In our study, we did not analyze the transcriptomic content of iPSC-derived secretome, which represents an important limitation as whole conditioned media was used to treat AC16 CMs. We did, however, identify proteins which can be further investigated as a therapeutic approach. Finally, little is known of the lipids in secretomes, and this field warrants further development. MSC-derived CM secretome lipids were suggested to have an anti-inflammatory role in an in vitro osteoarthritis chondrocyte model [68], but further investigation, specifically on the role of lipids in secretome, is needed to expand on the current research.

## 4. Materials and Methods

### 4.1. Generating Donor-Specific iPSC-Derived Secretome

Informed consent was obtained from donors prior to blood collection in accordance with McGill University Health Center Research Ethics Board approval. DCM patient criteria included the following: They must have been diagnosed with DCM based on the presence of enlargement and dilation of at least 1 ventricle and a left ventricular ejection fraction (LVEF) of less than 40%. A total of 3 DCM donors were then randomly selected and matched with 3 healthy controls having no cardiovascular diseases. Recruited patient information is summarized in Table 1. Line AIW001-02, a healthy donor line, was generously donated by Thomas Durcan and was generated via viral transfection methods [6,7].

Detailed iPSC transfection methods are provided in the Supplementary Materials, along with cell quality control methods. In brief, approximately 16 mL of peripheral blood was collected from each donor. CD34+ cells were isolated following a modified STEMCELL Technologies CD34+ isolation workflow. Isolated CD34+ cells were kept in liquid nitrogen until transfection. Thawed CD34+ cells having been expanded for 1 week underwent episomal transfection with Epi5™ Episomal iPSC Reprogramming Kit (Invitrogen™, Waltham, MA, USA) on the Neon™ Neon Transfection System with 3 electroporator pulses of 10 milliseconds at 1650 Volts. Cell culture was maintained in mTeSR™ Plus media (STEMCELL Technologies, Vancouver, BC, Canada). Generated iPSCs underwent quality control assessment (Figure S1) by staining for pluripotency markers, RT-qPCR (list of primers provided in Table S1), and trilineage differentiation. When storing cells for later use, iPSCs were frozen in CryoStor^®^ CS10 (STEMCELL Technologies).

Frozen iPSCs were thawed quickly at 37 °C before being diluted in mTeSR™ Plus media (STEMCELL Technologies), pelleted, and reconstituted in replating media consisting of mTeSR™ Plus media (STEMCELL Technologies) and 0.01 mM Y-27632 2HCl (ROCK inhibitor; Selleckchem, Cologne, Germany). Media was changed daily, and cells were passaged at least once before the secretome collection. When iPSCs were 90–100% confluent, cells were incubated with TeSR™-E8™ basal media (STEMCELL Technologies) for 24 h at 37 °C with 5% CO_2_. Conditioned media was collected and filtered through a 0.22 µm pore MF-MilliporeTM Membrane Filter (Sigma Aldrich, St. Louis, MO, USA). The secretome was stored at −20 °C until further use.

### 4.2. Mass Spectrometry

Lipids, detergents, and salts were removed via loading into stacking gel, which was then reduced with dithiothreitol, alkylated with iodoacetic acid, and digested with trypsin. Peptides re-solubilized in 0.1% aqueous formic acid were loaded onto an Acclaim Pepmap Easyspray (ThermoFisher Scientific, Waltham, MA, USA, 75 µM ID × 2 cm C18 3 µM beads) precolumn. Separation was then performed using analytical column separation using a Dionex Ulitimate 3000 uHPLC at 230 nL/min, with a gradient of 2–35% organic (0.1% formic acid in acetonitrile) over 3 h. Peptide analysis was performed via Thermo Orbitrap Fusion mass spectrometer (ThermoFisher Scientific, Waltham, MA, USA) at a 120,000 resolution (FWHM in MSI), with HCD sequencing (15,000 resolution) at the top speed for all peptides with a charge of 2+ or greater. Mascot 2.6.2 search engine (Matrix Science, Chicago, IL, USA) was used to search raw Mascot generic formatted data for human protein sequences (Uniprot, Cambridge, UK). Scaffold Q+ Scaffold_5.1.0 (Proteome Software, Portland, OR, USA) allowed for statistical treatment and data visualization.

### 4.3. IR Injury Modelling

AC16 immortalized CMs (Millipore Sigma, Burlington, MA, USA) were thawed and replated utilizing Dulbecco’s Modified Eagle Medium F12 (DMEM/F12; Gibco™, Waltham, MA, USA) with 10% fetal bovine serum (FBS; Gibco™) and 1% penicillin/streptomyocin (Gibco™). Once cells reached 90–100% confluency, AC16 CMs were replated for disease modelling at passages 9–13. Ischemia was simulated by placing cells in an air-tight container with an AnaeroPack (Mitsubishi Gas Chemical, Tokyo, Japan) for 24 h and incubated at 37 °C with DMEM/F12 media (Gibco™). Cells underwent reperfusion by completing a media change for incubation with iPSC-derived conditioned media from each donor-specific line or with media as a control and were incubated at 37 °C for 5 h reperfusion. Untreated controls were performed at the same time, with the only difference being that cells were placed in normoxia conditions for a 24 h period before reperfusion.

### 4.4. Resazurin Reduction Assay

Cells were plated on a 96-well plate with 6 replicates for each treatment condition. After reperfusion period, media was changed for 1:10 alamarBlue™ Cell Viability Reagent (Invitrogen™) in complete media for absorbance readings using a TECAN Infinite^©^ M200 Microplate reader (Tecan Group Ltd., Mennedorf, Switzerland) at 2, 3, and 4 h post-reperfusion. Resazurin reduction as an indicator for metabolic activity was calculated using the following formula:

$$\mathrm{Resazurin}\;\mathrm{reduction}\;(\%)=\frac{\left(117.216\right)\times A570-\left(80.586\right)\times A600}{\left(155.677\right)\times A^{\prime }600-\left(14.652\right)\times A^{\prime }570}\times 100\%$$

where *A*570: absorbance of test wells at 570 nm; *A*600: absorbance of test wells at 600 nm; *A*′600: absorbance of normoxia control wells at 600 nm; and *A*′570: absorbance of normoxia control wells at 570 nm.

### 4.5. Cell Viability

The same plating strategy used in the resazurin reduction assay was used for the Crystal Violet assay. Viability was measured by fixing cells with 4% paraformaldehyde (Electron Microscopy Sciences, Hatfield, PA, USA) and dyeing with a crystal violet staining reagent (Sigma Aldrich). Crystal violet was then solubilized using methanol, and absorbance readings were measured using the TECAN Infinite^©^ M200 Microplate reader (Tecan Group Ltd.). Optical density readings were adjusted by subtracting the mean reading of 3 blank wells from each value obtained. Viability percentage using the adjusted values was estimated according to the following formula:

$$\mathrm{Viability}(\%)=\frac{{OD}_{sample}}{{Mean\;OD}_{Normoxic\;Control}}\times 100\%$$

where *Mean OD* is the optical density at 540 nm. Once the resazurin reduction assay and cell viability confirmed that the amount of secretome utilized led to a response in AC16 cardiomyocytes, we proceeded with the rest of the experiments detailed below.

### 4.6. Hypertrophy Assessment

Untreated and treated cells plated in 24-well plates were fixed with 4% paraformaldehyde before being incubated with ActinRed™ 555 Ready Probes™ Reagent (Invitrogen™) and NucBlue™ Live ReadyProbes™ Reagent (Invitrogen™) as per the manufacturer’s instructions. Representative images were taken on a TH4-100 fluorescence microscope (Olympus Corporation, Tokyo, Japan), and cell area was measured using ImageJ imaging software version 1.54d (*n* > 28 cells per group).

### 4.7. Calcium Flux Analysis

AC16 CMs were seeded into 1.77 cm^2^ glass bottom dishes (CELLTREAT Scientific, Pepperell, MA, USA). Intracellular calcium was stained using 2.5 µM Fluo-4, AM, cell permeant (Invitrogen™) in DMEM/F12 (Gibco™) with 10% FBS (Gibco™) and 1% penicillin/streptomyocin (Gibco™) for 1 h. Media was changed for FluoroBrite™ DMEM (Gibco™) media for an additional 30 min before representative recordings of spontaneous intracellular calcium flux in live cells were made on a Zeiss LSM 780 confocal microscope (Zeiss, Oberkochen, Germany) and representative cells, approximately 28–101 per condition, were selected. Fluorescence intensity was measured in the selected cell at every 5 s from the beginning of the recording for 40 s. Obtained values were normalized to the mean of the normoxia group. For change in fluorescence intensity, the absolute difference between 2 time points taken 5 s apart for approximately 45 s we measured. The difference in fluorescence intensity was then normalized to the mean of the normoxia group.

### 4.8. Quantitative Reverse Transcription Polymerase Chain Reaction

For the quantitative reverse transcription polymerase chain reaction experiment, 1 million AC16 CMs were plated for each condition. The following day, cells underwent injury modelling as previously described, at the end of which RNA was collected using a RNeasy Plus Kit (QUIAGEN, Hilden, Germany) as per the manufacturer’s instructions with direct lysis of cells. RNA was frozen and kept at −80 °C until further use. Upon thawing, cDNA synthesis was performed using the iScript Advanced cDNA Synthesis Kit for RT-qPCR (Bio-Rad, Hercules, CA, USA) according to the manufacturer’s instructions. “No template” and “no reverse transcriptase” controls were also performed and added to each run. Obtained cDNA was then diluted in RNAse-free water and stored at −20 °C. Quantitative reverse transcription polymerase chain reaction was performed for each of the samples using the primers described in Table 2. Additionally, a standard curve was created utilizing a pooled sample and 1:2 serial dilution. Primers and samples were incubated with PowerUp™ SYBR™ Green Master Mix for RT-qPCR (Applied Biosystem™, Waltham, MA, USA) for 40 amplification cycles in the 7500 Real-Time PCR System (Applied Biosystems). The means of each technical replicate were used for subsequent analysis, and relative transcript abundance was obtained by normalization against the mean of the normoxia control for further analysis. Missing values were assumed to be 0.

### 4.9. Statistical Analysis

*t*-tests were used to determine statistical significance of differentially expressed proteins in iPSC secretome content from healthy or diseased donors. STRING was used to perform pathway analysis [33]. Outliers were identified and removed from analysis using the 1.5IQR method prior to one-way ANOVAs to determine significance between groups. Tukey’s multiple comparisons test was used to determine statistical significance between groups. All data are presented as mean ± SD with a significance level of *p* < 0.05. Graphs were generated using BioRender.

## 5. Conclusions

Secretome studies play an important role in the field of cardiovascular regeneration. Our findings contribute to the evolution of the regenerative field in three ways. First, our results further support the validity of iPSCs as a cell type for disease modelling. We were able to transfect and validate iPSCs from both healthy and disease patients from minimally invasive techniques, and these cells have different secretome contents based on donor health. This supports previous studies that have found iPSCs to conserve donor characteristics. Next, the differences in protein content of secretome content may explain some of the variation seen in the outcomes of clinical trials. As previously mentioned, many clinical trials have attributed observed benefits to the stem cell secretome rather than the cells themselves [15,16,17,18,19,20,21,22,23,24,25], and analyses of cell secretome load may aid in furthering our understanding and interpretation of results. To this effect, our findings also call for researchers to question the goal of proposed secretome treatments carefully. Long has been the debate of autologous versus heterologous donors for stem cell treatments, which may ultimately be a question of timing.

Indeed, iPSC-derived secretomes from healthy donors and DCM patients had no significant differences in protein levels involved in cell survival. These findings were supported by the observed increases in cell viability and survival, contributing to the growing evidence that the secretome is important in regenerative therapies. However, a healthy donor-derived iPSC secretome had increased concentrations of proteins involved in mitochondrial calcium homeostasis and calcium contractility. We found that donor health status can have an impact on therapeutic outcomes, as evidenced by increases in mean calcium fluorescence intensity observed in AC16 CMs with treatment of healthy donor-derived secretomes in the IR injury model, and further investigation into other important donor characteristics is warranted. Additionally, we evaluated the relative abundance of several transcripts involved in cell survival and calcium homeostasis and contractility but did not find any statistically significant differences. Other cellular and molecular mechanisms of action can be studied in the future to gain a meaningful understanding of the pathways utilized by EVs to effect change. For clinical translatability, we believe that these results highlight the impact that donor status can have on the quality of cell-free therapies, making it an important factor to consider as the field of regenerative therapy advances.

## Figures and Tables

**Table 1:** Summary of donor characteristics for each of the iPSC lines.

Sample ID	Age (Years)	Sex	Ethnicity	Healthy or DCM
HID041004	46	Female	Caucasian	Healthy
HID041032	55	Male	Black, other	Healthy
AIW001-02	48	Female	Caucasian	Healthy
HID041019	41	Female	Caucasian	DCM
HID041021	45	Female	Caucasian	DCM
HID041031	39	Male	Asian (East or Southeast Asian)	DCM
Average	45.66	66% (F)	-	50% (DCM)

**Table 2:** List of primers and their respective sequences used for RNA analysis of AC16 cells following ischemia–reperfusion injury model.

Gene Name	Abbreviation	Forward Sequence	Reverse Sequence
Hypoxia inducible factor 1 subunit alpha	*HIF1A*	TATGAGCCAGAAGAACTTTTAGGC	CACCTCTTTTGGCAAGCATCCTG
Superoxide dismutase 1	*SOD1*	TTTCGAGCAGAAGGAAAGTAATGGA	CAACATGCCTCTCTTCATCCTTTGG
Superoxide dismutase 2	*SOD2*	GATGTTACAGCCCAGATAGCTCTTC	CGTCAGCTTCTCCTTAAACTTGTCA
B-cell lymphoma 2	*BCL2*	AATTGCCGAGAAGGGGAAAACA	CGATTCCCAGACTTCTGCTTCA
B-cell lymphoma 2-associated X	*Bax*	GTTTTCTGACGGCAACTTCAACTG	AATGTCCAGCCCATGATGGTTC
Caspase-3	*CASP3*	GGAAGCGAATCAATGGACTCTGG	GCATCGACATCTGTACCAGACC
Atrial natriuretic peptide	*ANP*	GGTTCTGGTTGCCTTGGTAGGA	CCATGGCAACAAGATGACACAAATG
B-type natriuretic peptide	*BNP*	TCTGATCGATCTGCCCTCCTAAAAA	CAGGGTGTAGAGGACCATTTTGC
Sarcoplasmic/endoplasmic reticulum Ca^2+^ ATPase 2a	*SERCA2a*	GGACTTTGAAGGCGTGGATTGTG	CTCAGCAAGGACTGGTTTTCGG
Cardiac troponin T	*TNNT2*	GGAGGAGTCCAAACCAAAGCC	TCAAAGTCCACTCTCTCTCCATC
Calcium voltage-gated channel subunit Alpha1C	*CACNA1C*	TGATTCCAACGCCACCAATTC	GAGGAGTCCATAGGCGATTACT
Connexin 43	*Cx43*	CAATCTCTCATGTGCGCTTCT	GGCAACCTTGAGTTCTTCCTCT
Ryanodine receptor 2	*RYR2*	CATCGAACACTCCTCTACGGA	GGACACGCTAACTAAGATGAGGT
Solute carrier family 8 member A1	*NCX1*	CATTAAGAAGACAAACCTGGCCCTT	ACATTCATCGTCGTCATCATCTTCC

## Data Availability

Data are available upon request.

## Supplementary Materials

The following supporting information can be downloaded at: https://www.mdpi.com/article/10.3390/ijms251910279/s1.

## Abbreviations

Abbreviation	Definition
iPSC	Induced pluripotent stem cell
CM	Cardiomyocytes
MSC	Mesenchymal stem cell
RNA	Ribonucleic acid
DCM	Dilated cardiomyopathy
IR	Ischemia–reperfusion
LVEF	Left ventricular ejection fraction
DMEM/F12	Dulbecco’s modified eagle medium F12
FBS	Fetal bovine serum
ROS	Reactive oxygen species
HIF1A	Hypoxia inducible factor 1subunit alpha
SOD	Superoxide dismutase
BCL2	B-cell lymphoma 2
BAX	B-cell lymphoma 2-associated X
CASP3	Caspase 3
ANP	Atrial natriuretic peptide
BNP	Brain natriuretic peptide
SERCA2A	Sarcoplasmic/endoplasmic reticulum Ca^2+^ ATPase 2a
TNNT2	Cardiac troponin T
CACNA1C	Calcium voltage-gated channel subunit alpha 1 C
CNX43	Connexin 43
RYR2	Ryanodine receptor 2
NCX1	Sodium/calcium exchanger 1
