# Supplementary material for: Evaluating the Reparative Potential of Secretome from Patient-Derived Induced Pluripotent Stem Cells during Ischemia–Reperfusion Injury in Human Cardiomyocytes
Source: Int J Mol Sci. 2024 Sep 24;25(19):10279. doi: 10.3390/ijms251910279 (PMC11477076; doi:10.3390/ijms251910279)
Supplement: Supplementary file 1 [file ijms-25-10279-s001.zip › ijms-3159307-supplementary.pdf]

Supplementary Materials

Methods:

Approximately 16 ml of peripheral blood was obtained and kept at 4°C until CD34+ isolation. Processing of samples occurred within 12 hours of collection and largely followed the STEMCELL Technologies CD34+ isolation workflow with a few modifications. Briefly, peripheral blood mononuclear cells were isolated by diluting whole blood with EasySep™ dilution buffer (STEMCELL Technologies). Diluted blood was carefully layered on top of the Lymphoprep™ (STEMCELL Technologies) so as to maintain phase separation. Tubes were centrifuged to obtain density gradient before placing supernatant in new conical tube and centrifuging cells again to pellet cells. Cell pellets were resuspended in EasySep™ buffer (STEMCELL Technologies) before performing the CD34+ positive selection (EasySep™ buffer, STEMCELL Technologies). Isolated CD34+ cells were then diluted in fetal bovine serum (FBS; Gibco™) with approximately 12% dimethyl sulfoxide (DMSO; Sigma Aldrich) for cryopreservation and kept at <-150°C for long-term storage.

A week before transfection, isolated CD34+ cells were thawed by diluting freezing medium in StemSpan™ SFEM II with StemSpan™ CC100 (STEMCELL Technologies) and centrifuging to pellet cells. Buffered DNAse I (Thermo Scientific™) was subsequently added to cell pellet and incubated for 15 minutes at room temperature. Media was topped up to 2mL and plated in one well of a 6-well plate and cells were incubated at 37°C with 5% CO_2. Complete media changes were performed every other day for a total of 3 media changes. On the day of transfection, CD34+ cells were collected, pelleted by centrifugation, washed with Dulbecco's phosphate buffered saline (Sigma Aldrich) before final reconstitution in Dulbecco's phosphate buffered saline (Sigma Aldrich). Epi5™ Episomal iPSC Reprogramming Kit (Invitrogen™) was used as per manufacturer's instruction with 3 electroporator pulse of 10 milliseconds at 1650 Volts (Neon™ Neon Transfection System). Electroporated cells were reconstituted in StemSpan™ SFEM II with StemSpan™ CC100 (STEMCELL Technologies) and plated on Matrigel-coated (Corning®) 6-well plates.

The following day, StemSpan™ SFEM II with StemSpan™ CC100 (STEMCELL Technologies) was added to each well. The next day TeSR™-E7™/ReproTeSR™ (STEMCELL Technologies) media was added to each well. Two days later, daily complete media changes were performed substituting old media for TeSR™-E7™/ReproTeSR™ (STEMCELL Technologies) until establishment of iPSC colonies. Daily media changes were then performed utilizing mTeSR™ Plus media (STEMCELL Technologies). Colonies were passaged mechanically by manually selecting colonies for replating until P4 and subsequently passaged at 80% or higher confluence via chemical dissociation utilizing Gentle Cell Dissociation Reagent (STEMCELL Technologies). In both cases, iPSCs were replated in replating media constituted of mTeSR™ Plus media (STEMCELL Technologies) with Y-27632 2HCl (ROCK inhibitor; Selleckchem). Cells were frozen by reconstituting cell pellets in CryoStor® CS10 (STEMCELL Technologies) and stored at <-150°C for long-term storage. IPSC line AIW001-02 was generously donated by Thomas Durkan and underwent viral transfection method (1, 2).

Newly generated iPSCs were validated by immunocytochemistry staining of pluripotency markers with later confirmation via quantitative reverse transcription polymerase chain reaction, trilineage differentiation and karyotyping. Briefly, iPSCs were fixed and stained using POU class 5 homebox 1 (OCT3/4; Cell Signaling Technology), stage-specific embryonic antigen 4 (SSEA4; Cell

Signaling Technology), nanog homeobox (NANOG; Cell Signaling Technology), and podocalixin like (TRA-1-60(S); Cell Signaling Technology). Cells were also incubated with ActinRed™ 555 Ready Probes™ Reagent (Invitrogen™) and NucBlue™ Live ReadyProbes™ Reagent (Invitrogen™) as per the manufacturer's instructions. Stains were visualized and at least 3 representative images of each stain were taken on a TH4-100 fluorescence microscope (Olympus Corporation). RNA was collected using Aurum™ Total RNA Mini Kit (Bio-Rad) as per manufacturer's spin protocol. RNA was frozen and kept at -80°C until further use. Upon thawing, cDNA synthesis was performed using the iScript Advanced cDNA Synthesis Kit for RT-qPCR (Bio-Rad) according to the manufacturer's instructions. Obtained cDNA was then diluted in RNAse-free water. Diluted cDNA stored at -20°C until further use. Quantitative reverse transcription polymerase chain reaction was performed for each of the samples using the primers described in **Table S1**. Relative transcript abundance was then normalized to glyceraldehyde 3-phosphate dehydrogenase (GAPDH). Trilineage differentiation potential was assessed using the Human Pluripotent Stem Cell Functional Identification Kit (R&D SYSTEMS) or the STEMdiff™ Trilineage Differentiation Kit (STEMCELL Technologies) according to manufacturer's instructions. Successful differentiation of Ectoderm, Mesoderm, Endoderm Cell lineages were performed by staining cells for orthodenticle homeobox 2 (OTX2; R&D SYSTEMS or Proteintech), Brachyury (R&D SYSTEMS or Invitrogen™), and SRY-box transcription factor 17 (SOX17; R&D SYSTEMS or Invitrogen™) respectively. Representative images were acquired using TH4-100 fluorescence microscope (Olympus Corporation

Table S1: List of Primers and their respective sequences used for PCR analysis of iPSC pluripotency markers

Gene	Abbreviation	Forward Sequence	Reverse Sequence
Glyceraldehyde 3-phosphate dehydrogenase	GAPDH	GAATGGGCAGCCGTTAGGAA	GACTCCACGACGTACTCAGC
POU class 5 homebox 1	OCT3/4	ACCCCTGGTGCCGTGAA	GGCTGAATACCTTCCCAAATA
Nanog homeobox	NANOG	AAAGGCAAACAACCCACT	GCTATTCTTCGGCCAGTT
SRY-box transcription factor 2	SOX2	CAGCGCATGGACAGTTAC	GGAGTGGGAGGAAGAGGT
Transforming growth beta superfamily of cytokines	TGFB	TCCTTCTACGGACGGAACTG	AGAAATGCCTGAGGAAAGCA
Lin-28 homolog A	LIN28	GTTCGGCTTCCTGTCCAT	CTGCCTCACCCTCCTTCA

Additional Results and Discussion:

We confirmed presence of pluripotency markers using OCT3/4, SSEA4, Nanog, and TRA-1-60(S) via immunocytochemistry (**Figure S1A**). All episomal-generated lines showed presence of markers. Additional confirmation was performed quantitative reverse transcription polymerase chain reaction and once again all lines confirmed presence of markers (**Figure S1B**). Pluripotency potential was further confirmed by trilineage differentiation of iPSCs into endodermal, mesodermal and ectodermal cell lineages (**Figure S1C**).

Figure S1: Example results from iPSC line validation. (A) Representative images of iPSCs following staining of pluripotency markers. (B) Example of quantitative reverse transcription polymerase chain reaction amplification plot and (C) results normalized to GAPDH following amplification of transcripts of genes associated with pluripotency. (D) Representative images of iPSCs having been differentiated into endodermal, mesodermal and ectodermal cell lineages followed by fixing and staining with OTX2, brachyury and SOX17 respectively. OCT3/4= POU class 5 homebox 1, SSEA4= stage-specific embryonic antigen 4, NANOG=nanog homeobox, TRA-1-60(S)=podocalixin like, GAPDH= Glyceraldehyde 3-phosphate dehydrogenase, SOX2= SRY-box transcription factor 2, TGFB= Transforming growth beta superfamily of cytokines, LIN28= Lin-28 homolog A

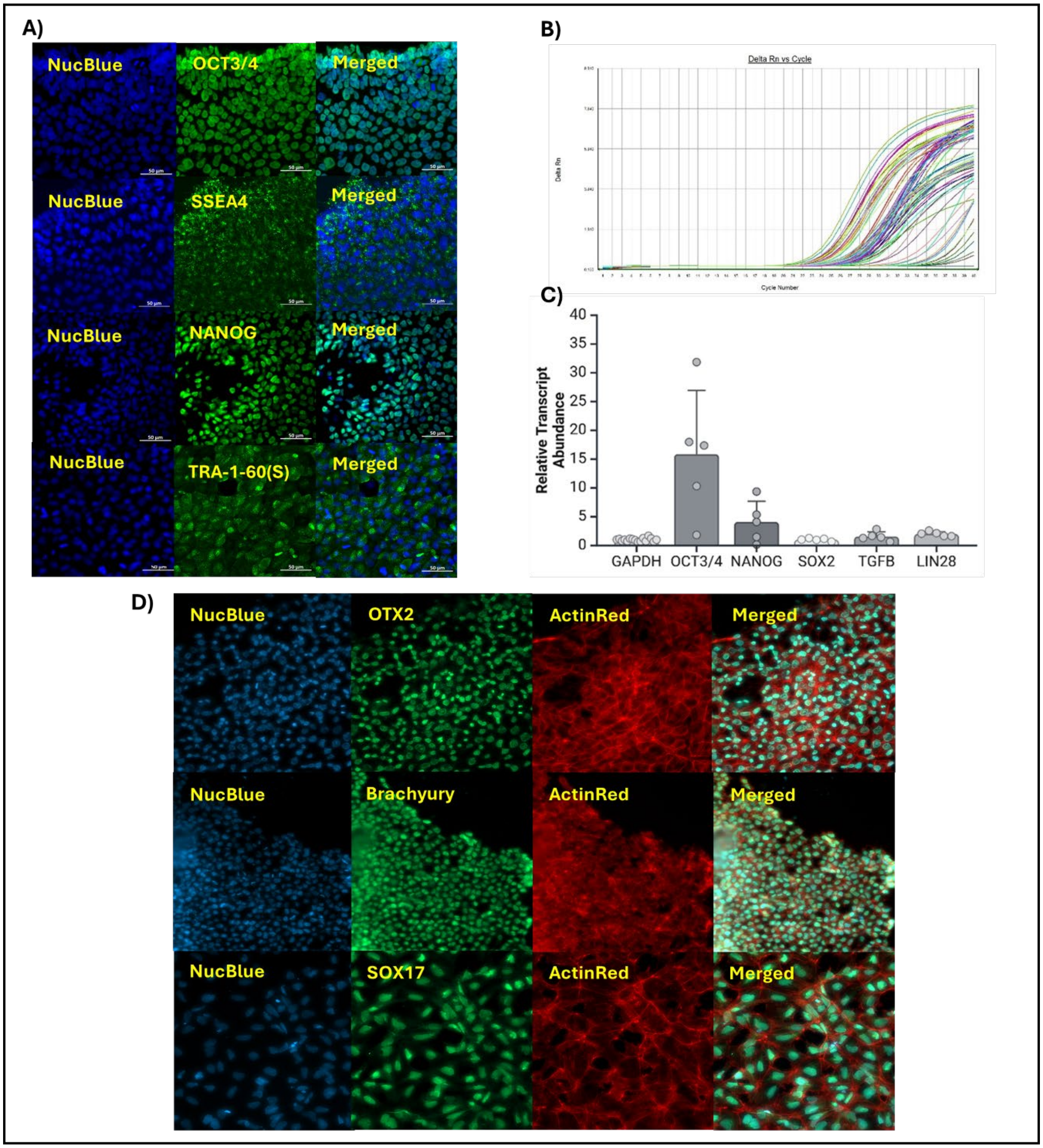

Reference List:

1. Wen W, Zhang JP, Xu J, Su RJ, Neises A, Ji GZ, et al. Enhanced Generation of Integration-free iPSCs from Human Adult Peripheral Blood Mononuclear Cells with an Optimal Combination of Episomal Vectors. Stem Cell Reports. 2016 Jun 14;6(6):873-884. doi: 10.1016/j.stemcr.2016.04.005.

2. Chen CX, Abdian N, Maussion G, Thomas RA, Demirova I, Cai E, et al. A Multistep Workflow to Evaluate Newly Generated iPSCs and Their Ability to Generate Different Cell Types. Methods and Protocols. 2021 Jul 19;4(3):50. doi: 10.3390/mps4030050.
